# Do we still accept central venous pressure measurements to assess preload responsiveness in children with septic shock? A single-center experience

**DOI:** 10.1186/cc11774

**Published:** 2012-11-14

**Authors:** K Sasidaran, M Jaishree, S Singhi

**Affiliations:** 1 PGIMER, Chandigarh, India

## Background

Rapid and timely fluid resuscitation is key to improved outcomes in septic shock. Hemodynamic monitoring is vital for preload assessment. Central venous pressure (CVP) is one such traditional tool that is currently falling out of favor as adult data have shown it to be less reliable in predicting preload responsiveness. Pediatric data are, however, limited. We conducted this study to assess the predictive utility of CVP in determining the preload responsive status defined as a stroke index increment (≥15%) after preload (20 ml/kg 0.9% saline) administration.

## Methods

A total of 166 episodes of preload administrations were included. Hemodynamic variables (heart rate, mean arterial blood pressure, CVP) were measured before (T0) and after (T1) preload administration in all. Both spontaneously breathing and mechanically ventilated children were enrolled. HR decrement and CVP increment as response to preload were calculated using T1 and T0 measurements. The stroke index was measured by transthoracic echocardiography at T0 and T1. Infants <1 month of age, improper position of central venous catheter tip, clinical evidence of increased intra-abdominal pressure, previously diagnosed heart disease, need for high-frequency ventilation, and children in whom family had declined consent were excluded from the study.

## Results

Of the 166 episodes, 120 (72%) were fluid responsive and 46 (28%) were nonresponsive. One hundred and twenty-five episodes occurred in patients on positive pressure ventilation and 41 during spontaneous breathing. The internal jugular vein was catheterized in 34 (142 episodes; 85%) and the femoral vein in seven patients (24 episodes; 14.5%). There was a significant decrease in heart rate independent of ventilator support in the preload responsive as compared with the nonresponsive group (24 ± 9 vs. 4 ± 7; *P *= 0.001). However, there was no significant difference in CVP between the fluid responsive and nonresponsive episodes (6.2 ± 1.5 vs. 6.0 ± 1.4; *P *= 0.71), although CVP was significantly higher in mechanical ventilation as compared with episodes on spontaneous breathing (6.5 ± 1.3 vs. 5.2 ± 1.6; *P *= 0.001). Baseline CVP (T0) (*r *= 0.01; *P *= 0.10) and CVP increment at T1 (*r *= 0.18; *P *= 0.14) showed a poor correlation with stroke index increment.

## Conclusion

CVP increment with preload showed a poor correlation with stroke index increment, suggesting that CVP measurements are not useful in predicting a preload responsiveness state in critically ill children. Decrement in heart rate was the only conventional hemodynamic variable that signified preload response but that also in retrospect, thus limiting its utility as a predictive tool.

